# Concurrent Eruptive Melanocytic Nevi and Multiple Keratoacanthomas Induced by Encorafenib

**DOI:** 10.7759/cureus.70540

**Published:** 2024-09-30

**Authors:** Nektarios Alevizopoulos, Dimitrios Alexandris, Michail G Pavlakis, Kypros Dimosthenous, Georgios Pavlakis

**Affiliations:** 1 Oncology, Evangelismos General Hospital, Athens, GRC; 2 Internal Medicine, Evangelismos General Hospital, Athens, GRC; 3 Oncology, Biokliniki Athinon, Athens, GRC; 4 Pathology, Metropolitan General Hospital, Athens, GRC

**Keywords:** adverse drug reaction reporting, braf-inhibitors, cutaneous adverse drug reaction, encorafenib, eruptive melanocytic nevi, multiple keratoacathomas, panitumumab

## Abstract

Eruptive melanocytic nevi and multiple keratoacanthomas are rare cutaneous conditions, often linked to drug-related toxicities but rarely reported simultaneously, particularly in cancer patients undergoing BRAF-targeted therapies. We present a case of a 64-year-old male with metastatic colorectal cancer who developed severe pruritus and widespread new skin lesions following treatment with encorafenib, a v-Raf murine sarcoma viral oncogene homolog B (BRAF) inhibitor, and panitumumab, an anti-epidermal growth factor receptor (EGFR) monoclonal antibody. The dermatological assessment identified both pigmented and non-pigmented lesions, including benign nevi and keratoacanthomas. Despite close monitoring, larger keratoacanthomas persisted, prompting surgical excision, which confirmed lichenoid keratosis. The Naranjo scale indicated a definite association between encorafenib and cutaneous reactions, with a score of nine. The mitogen-activated protein kinase pathway’s role in cutaneous adverse events was explored, suggesting a paradoxical activation mechanism. Discontinuation of encorafenib and panitumumab led to gradual resolution of most lesions, highlighting a possible etiopathogenic link. This study emphasizes the need for thorough dermatologic evaluation and follow-up in patients receiving BRAF inhibitors to optimize management and avoid unnecessary treatment cessation.

## Introduction

Eruptive melanocytic nevi (EMN) is a cutaneous condition characterized by the rapid appearance of numerous melanocytic nevi on the skin [1]. Although the exact mechanisms remain under investigation, EMN has been associated with immunodeficiency, bullous skin disorders, and drug-related toxicities [2]. Multiple keratoacanthomas (MK) present as numerous papules with central keratotic plugs and well-demarcated borders [3]. Persistent pruritus is rarely reported as a concurrent symptom [3]. Both EMN and MK have been infrequently described as drug-related toxicities in cancer patients treated with BRAF (v-Raf murine sarcoma viral oncogene homolog B)-targeted therapies but have not been documented to occur simultaneously at the onset of anti-BRAF medication. We present a case of a 64-year-old male with metastatic colorectal cancer who developed simultaneous eruptive melanocytic nevi and multiple keratoacanthomas following treatment with encorafenib and panitumumab.

## Case presentation

A 64-year-old male was diagnosed with metastatic colorectal cancer. Based on his molecular profile, which was wild type for Kirsten rat sarcoma viral oncogene homolog (KRAS)/neuroblastoma rat sarcoma viral oncogene homolog (NRAS) and positive for a BRAF mutation, he was initially treated with folinic acid, fluorouracil, and oxaliplatin (FOLFOX) and cetuximab as a first-line regimen. However, his metastatic liver disease did not respond, and progressive disease was documented. Consequently, he was switched to a second-line regimen of encorafenib, an oral anti-BRAF inhibitor, in combination with panitumumab, an anti-epidermal growth factor receptor (EGFR) monoclonal antibody. Two months after starting this treatment, the patient reported severe pruritus that was not adequately managed by antihistamines. The dermatological evaluation revealed widespread new skin lesions.

The patient’s medical history was notable only for psoriasis, well-controlled with topical steroids. There was no history of skin tumors. A thorough dermatological assessment was performed, including full-body photographic documentation and dermoscopic examination of all lesions to exclude other dermatological conditions. We identified multiple melanocytic and non-melanocytic lesions with well-demarcated borders on the patient’s trunk, face, scalp, extremities, palms, and soles.

**Figure 1:**
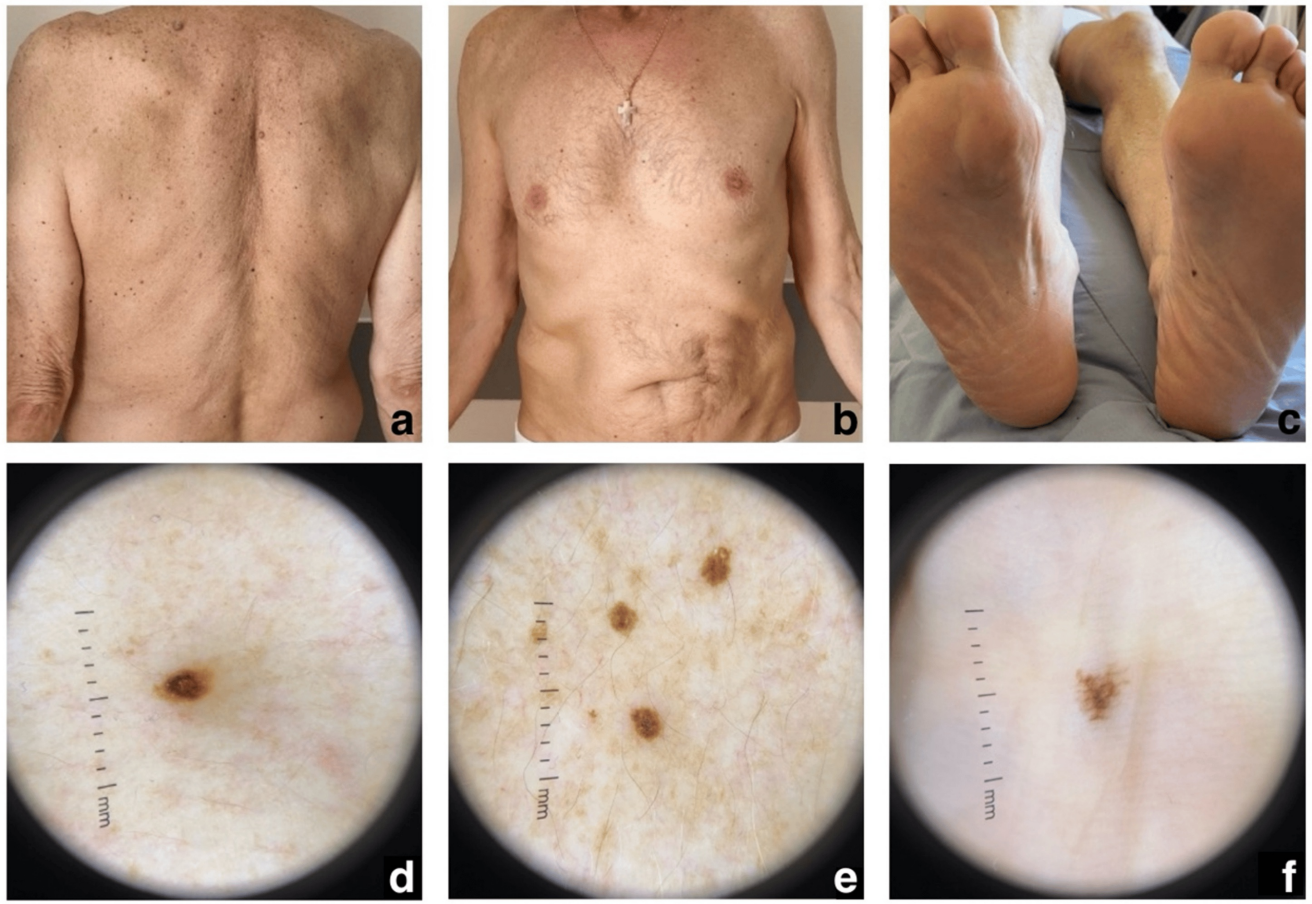
Clinical and dermoscopic examination images of the patient. Clinical images of the patient’s body and soles (A-C). Dermoscopic examination of these lesions showed benign nevi with central hyperpigmentation (D), a homogeneous pattern (E), and a parallel furrow pattern (F).

New pigmented lesions were identified as benign nevi. Dermoscopic criteria included a network pattern with a darker center or amorphous areas, similar to the patient’s pre-existing nevi, while nevi on the palms and soles displayed parallel furrow, lattice, or homogeneous patterns. The patient reported no changes in his existing nevi’s shape, size, or color. All nevi remained stable during a three-month follow-up period, ruling out other underlying clinical entities (Figures 1a-1f).

**Figure 2:**
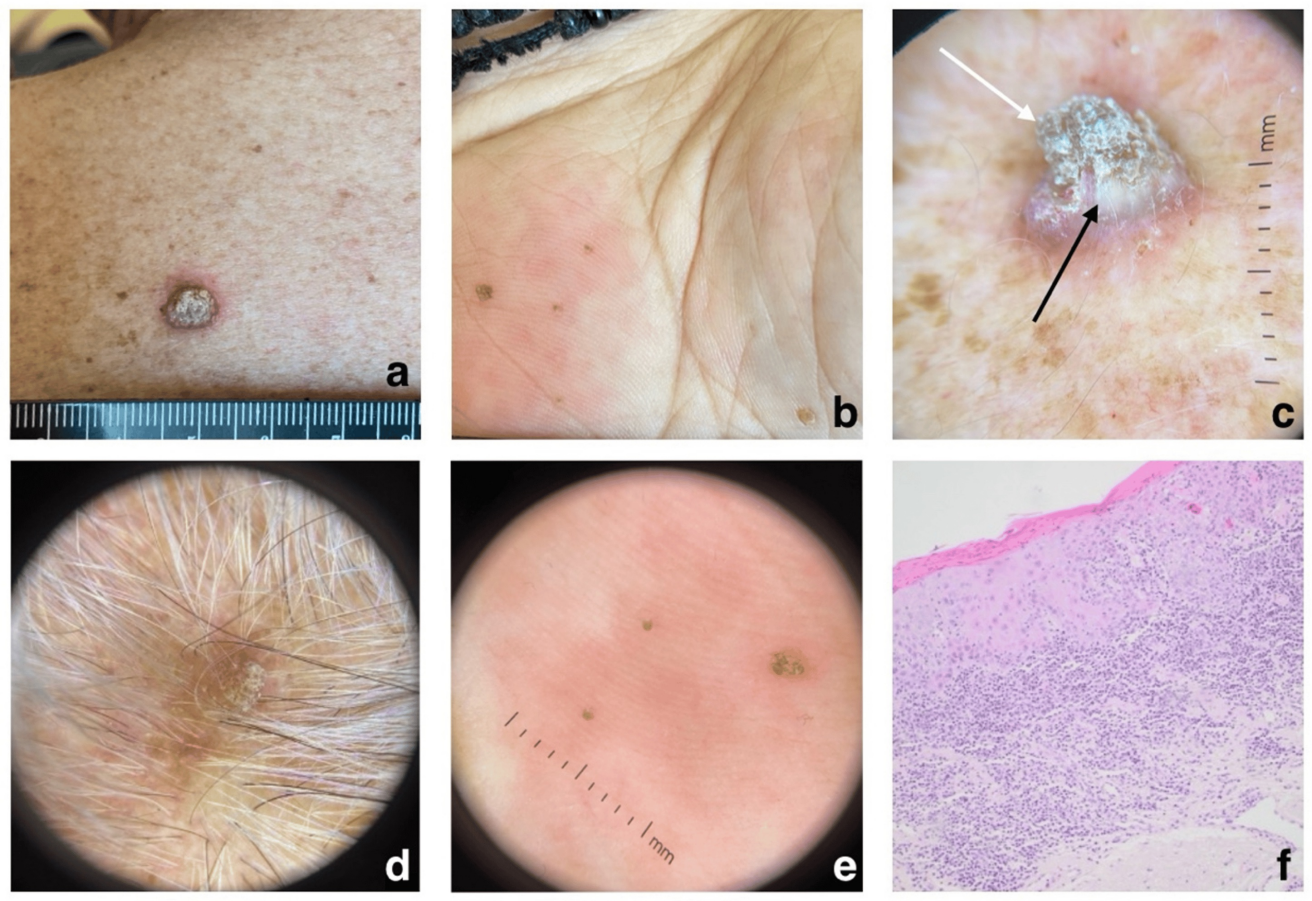
Dermoscopy revealed features suggestive of keratoacanthomas. Macroscopic images of the patient’s keratoacanthomas on the body (A, B), with dermoscopic images of the lesions on the body (C), scalp (D), and palms (E). Dermoscopy of lesion C reveals a white background, hairpin-like vessels at the periphery (black arrow), and a central keratotic plug (white arrow). Histologic image of lichen keratosis four months after treatment cessation shows parakeratosis, acanthosis, civatte bodies in the epidermis, and a dense lichenoid lymphocytic infiltrate (F).

Non-pigmented lesions appeared as well-demarcated papules with central keratotic plugs located on sun-exposed skin, extremities, and palms. The patient reported severe pruritus across the body. The dermoscopic examination suggested the presence of keratoacanthomas (Figures 2a-2f).

The patient did not respond to the encorafenib and panitumumab combination beyond five months of treatment, with new metastatic liver lesions indicating disease progression. The dual-targeted therapy was discontinued. Notably, many newly identified pigmented and non-pigmented lesions gradually resolved with ongoing monitoring, except for larger keratoacanthomas, which remained stable. To rule out squamous cell carcinoma, his care team surgically excised the larger lesions, and histological evaluation confirmed the diagnosis of lichenoid keratosis.

We used the Naranjo scale to determine the likelihood of drug-related adverse events. In this case, the score was nine, indicating that the skin reactions were definitely induced by encorafenib. The prior cetuximab treatment in the first-line regimen with FOLFOX was unlikely to have caused these cutaneous manifestations. Encorafenib, particularly when combined with another anti-EGFR monoclonal antibody, is more commonly associated with such cutaneous reactions [4].

## Discussion

The mitogen-activated protein kinase (MAPK) pathway is a crucial signaling pathway that regulates various cellular functions, including keratinocyte differentiation and melanocyte proliferation [2,5]. Encorafenib, a selective, adenosine triphosphate-competitive BRAF inhibitor, is used as a targeted therapy in many malignancies [5]. Dummer et al. extensively documented cutaneous adverse events associated with encorafenib, including rash, hyperkeratosis, cutaneous squamous cell carcinoma, keratoacanthomas, basal cell carcinoma, and photosensitivity reactions [6], with EMN reported as a rare occurrence [5-7].

The MAPK pathway plays a critical role in developing cutaneous adverse events. One theory suggests that dimer formation between rapidly accelerated fibrosarcoma (RAF) molecules in BRAF wild-type cells can paradoxically activate the MAPK pathway, potentially contributing to these skin reactions [7]. The widespread impact of MAPK kinase on various cutaneous functions underscores its possible involvement in adverse events associated with targeted therapies [8].

Identifying drug-related adverse reactions is essential to understanding their underlying mechanisms. The Naranjo adverse drug reaction probability scale is a commonly used tool to assess the likelihood of a drug causing an adverse event [4]. This scale involves answering 10 questions about the suspected medication and the reaction, with each response contributing to a total score. The final score categorizes the association as unlikely, possible, probable, or definite. In this case, the Naranjo scale indicated a definite relationship between encorafenib use and the development of both MK and EMN. Notably, panitumumab, an anti-EGFR monoclonal antibody given in combination with encorafenib, was used as part of the first-line regimen without similar adverse events (Table 1) [4].

**Table 1:** Results of Naranjo adverse drug reaction probability scale. MEN: multiple eruptive nevi; MK: multiple keratoacanthomas

No.	Naranjo questions	MEN	MK
1.	Are there previous conclusive reports on this reaction?	1	1
2.	Did the adverse event occur after the suspected drug was administrated?	2	2
3.	Did the adverse reaction improve when the drug was discontinued or a specific antagonist was administered?	1	1
4.	Did the adverse reaction reappear when the drug was readministered?	2	2
5.	Are there alternative causes (other than the drug) that could have on their own cause the reaction?	2	2
6.	Did the reaction reappear when the placebo was given?	0	0
7.	Was the drug detected in the blood (or other fluids) in concentrations known to be toxic?	0	0
8.	Was the reaction more severe when the dose was increased or less severe when the dose was decreased?	0	0
9.	Did the patient have a similar reaction to the same or similar drugs in any previous exposure?	0	0
10.	Was the adverse event confirmed by any objective evidence?	1	1
Total score		9	9

Both adverse events are rarely reported, and no specific management guidelines exist. The patient’s lesions were closely monitored dermoscopically to detect any changes promptly. During the initial three-month follow-up, no significant alterations were observed. Upon cessation of encorafenib and panitumumab due to progressive disease, the adverse events gradually resolved, suggesting a potential etiopathogenic link.

To our knowledge, MK and EMN are rarely reported in patients treated with encorafenib [5,6]. Limited data exist regarding the risk of skin cancer in patients experiencing BRAF inhibitor-induced cutaneous adverse events. Therefore, precise dermatologic evaluation and adequate follow-up are recommended to reduce morbidity and mortality and prevent premature discontinuation of cancer therapies.

## Conclusions

Many questions remain regarding the pathophysiology of these unusual adverse events. Thorough dermatologic evaluation and histological confirmation are strongly recommended to ensure evidence-based management. This approach will help physicians optimize patient outcomes with novel targeted therapies, minimizing unnecessary treatment discontinuation.
